# Bitter Almond Albumin ACE-Inhibitory Peptides: Purification, Screening, and Characterization In Silico, Action Mechanisms, Antihypertensive Effect In Vivo, and Stability

**DOI:** 10.3390/molecules28166002

**Published:** 2023-08-10

**Authors:** Nan Qin, Chao Chen, Najun Zhang, Lulu Song, Yunfei Li, Lili Guo, Rui Liu, Wenfang Zhang

**Affiliations:** College of Medicine and Food Engineering, Shanxi University of Chinese Medicine, Taiyuan 030619, China; chenhanchen2010@163.com (C.C.); zhangnajun2747@163.com (N.Z.); 18768942280@163.com (L.S.); 15670138865@163.com (Y.L.); cauguolili@163.com (L.G.); ruiliu87@126.com (R.L.); m18935412709@163.com (W.Z.)

**Keywords:** bitter almond albumin peptides, inhibitory mechanisms against ACE, docking mode, zinc coordination patterns, antihypertensive effect

## Abstract

Almond expeller is an undeveloped reservoir of bioactive peptides. In the current study, a zinc ion ligand Arg-Pro-Pro-Ser-Glu-Asp-Glu-Asp-Gln-Glu (RPPSEDEDQE) offering a noncompetitive inhibitory effect on ACE (IC_50_: 205.50 μmol·L^−1^) was identified from almond albumin hydrolysates via papain and thermolysin hydrolysis, subsequent chromatographic separation, and UPLC-Q-TOF-MS/MS analysis. Molecular docking simulated the binding modes of RPPSEDEDQE to ACE and showed the formation of hydrogen bonds between RPPSEDEDQE and seven active residues of ACE. Moreover, RPPSEDEDQE could bind to fifteen active sites of ACE by hydrophobic interactions, and link with the His387 and zinc ions of the zinc tetrahedral coordination. Ultraviolet wavelength scanning and Fourier-transformed infrared spectroscopy analysis revealed that RPPSEDEDQE can provide multiple binding sites for zinc ions. However, RPPSEDEDQE cannot bind with any central pocket of ACE, which was evidenced by an inhibition kinetics experiment. Additionally, the zinc-chelating capacity and inhibiting ability against ACE of RPPSEDEDQE were both not significantly reduced by the hydrolysis of gastrointestinal enzymes. A moderate to high dose of RPPSEDEDQE (100–150 mg·kg bw^−1^) significantly reduced the systolic and diastolic blood pressure of spontaneous hypertensive rats, but chelation with zinc ions decreased its antihypertensive efficiency. These results indicate that bitter almond albumin peptides may be used for lowering blood pressure.

## 1. Introduction

Nowadays, natural antihypertensive peptides have received increasing attention, because approximately 10.8 million people worldwide die from hypertension complications every year, and synthesized antihypertensive drugs usually have side effects such as vomiting, coughing, allergies, and loss of appetite [1]. In comparison, antihypertensive peptides from foods are considered to be more efficient and safe [2]. The inhibition effect on the ACE-Ang II-AT1R axis has been shown to be the main blood-pressure-lowering pathway of antihypertensive drugs [3]. In the ACE-Ang II-AT1R axis, Angiotensin-I-Converting Enzyme (ACE) is found to play a crucial role in the elevation of blood pressure. Previous studies evidenced that the binding center of ACE contained S1, S2, and S′ central pockets that consisted of nine amino acid residues (Lys511, Tyr523, His353, Glu384, His513, Ala354, Glu162, Gln281, and Tyr520), where its catalytic center contained a zinc tetrahedral coordination that consisted of a zinc ion linked with Glu411, His383, and His387 residues [4,5]. Inhibitors of strong binding power with one or more of the nine active sites in the ACE central pocket are found to be competitive inhibitors of ACE. Moreover, peptides that can affect the structure of ACE via binding with other active sites outside the central pockets are shown to offer inhibition activity toward ACE [6]. Additionally, the zinc ions located in the zinc tetrahedral ligand are essential for the catalytic action of ACE [2]. Thus, in theory, peptides that can influence the zinc tetrahedral coordination of ACE should have a potential inhibition capacity against ACE [7]. In the last decades, peptides with inhibition activity against ACE have been extensively isolated from food protein resources [5,8,9]. However, to our best knowledge, evidence that peptides with zinc-chelating ability can inhibit the activity of ACE is currently weak [2].

In addition, substantial evidence suggests that antihypertensive peptides derived from food proteins may be sensitive to digestion and food processing conditions, especially gastrointestinal digestion [10]. Pepsin, trypsin, and other enzymes presenting in the stomach or intestines can change the amino acid sequence of peptides, further cause changes in the electrochemical parameters of peptides, thereby affecting the coordination of peptides with ACE or zinc ions, and lowering their inhibitory ability toward ACE [11,12,13]. However, few studies offer the effect of gastrointestinal digestion on ACE-inhibitory activity, physicochemical parameters, coordination with zinc ions, and stability of peptides.

Nuts are an important reservoir of antihypertensive peptides. Recently, ACE-inhibitory peptides have been isolated from different nut proteins such as walnut protein, pine nut globulin, almond, and hazelnut protein isolates [14,15,16,17]. Almond (*Semen Armeniacae Amarum*) has a high content of protein (around 37 g·100 g^−1^) [18]. The annual production of almond increased with an increasing worldwide almond yield and demand for apricot oil [19]. Bitter almond protein has a relatively desirable amino acid composition and high emulsifying properties, foam, and gelling properties [20,21,22]. Bioactive peptides isolated from bitter almond protein hydrolysates were shown to offer health-care functions such as a hypoglycemic effect, antihypertension, and antiradiation activity [23,24]. Zhang & Ye identified a pentapeptide with a high zinc chelating ability from sweet almond expeller amandin hydrolysates [25]. Mirzapour et al. [16] isolated ACE-inhibitory peptides from wild almond proteins. However, there is little data simultaneously referring to the ACE-inhibitory activity and zinc-chelating ability of bitter almond peptides. Our pre-experimental showed that the zinc-chelating ability and inhibition ability toward ACE of bitter almond albumin hydrolysates (BAAHs) were 20.67 ± 3.58 mg·g^−1^ and 54.95 ± 4.62%, respectively, indicating that peptides possessing zinc-chelating and ACE-inhibition capacities should be isolated from BAAHs. To improve the antihypertensive function of BAAHs, the aim of this study was to study the isolation, characterization, action mechanism, and zinc ion-chelating capacity of BAAH ACE-inhibitory peptides. Additionally, the physicochemical characteristics, antihypertensive effect in vivo, and stability of BAAH ACE-inhibitory peptides were investigated.

## 2. Results

### 2.1. Chromatographic Purification of BAAHs

**Figure 1:**
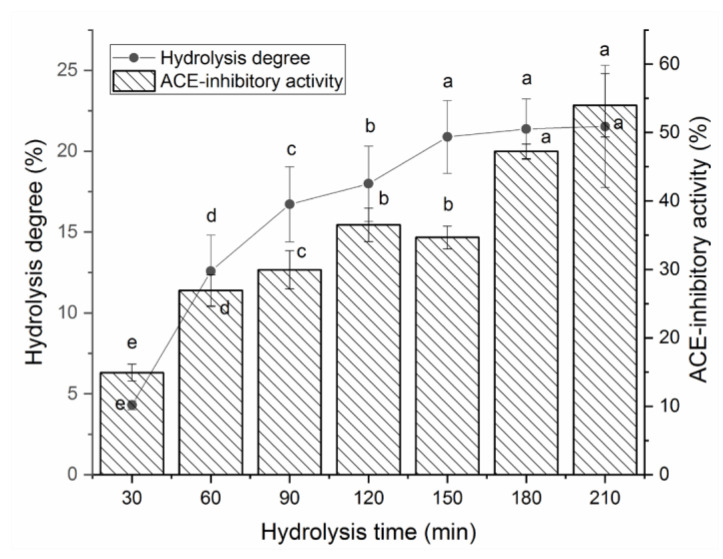
The hydrolysis degree and ACE-inhibitory activity of almond albumin hydrolysates at different hydrolysis times. Different letters (a–e) between the groups marked a statistically significant difference (*p* < 0.05).

Figure 1 shows the effects of papain and thermolysin on the hydrolysis degree and ACE-inhibitory activity of BAAHs with an increasing hydrolysis time. From 30 to 150 min, both the hydrolysis degree and ACE-inhibitory activity of BAAHs were increased as the hydrolysis time increased. After 180 min, the hydrolysis degree and ACE-inhibitory activity of BAAHs were both not significantly increased with the increasing hydrolysis time. After hydrolysis for 210 min, the hydrolysis degree and ACE-inhibitory activity of BAAHs were 21.53 ± 5.78% and 54.95 ± 4.62% (Figure 1), respectively.

**Figure 2:**
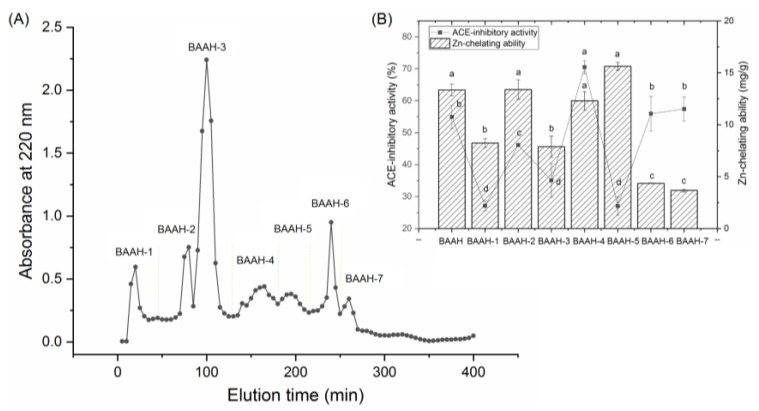
(**A**) The chromatographic separation diagram of almond albumin hydrolysates (BAAHs) on Sephadex G-15 gel column, and (**B**) zinc ion-chelating ability and inhibiting capacity against ACE of the separated main peaks separately named as BAAH-1, BAAH-2, BAAH-3, BAAH-4, BAAH-5, BAAH-6, and BAAH-7. Different letters (a–d) between the groups marked a statistically significant difference (*p* < 0.05).

**Figure 3:**
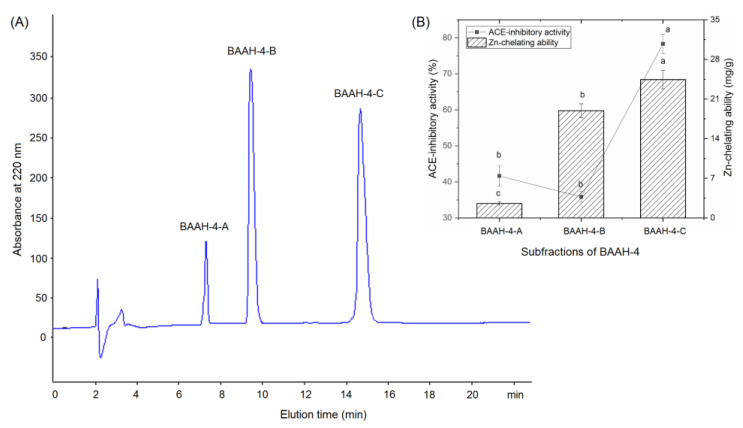
(**A**) The chromatographic separation diagram of BAAH-4 in reverse-phase high-performance liquid chromatography system, and (**B**) the zinc ion-chelating ability and inhibiting capacity against ACE of the obtained main subfractions named as BAAH-4-A, BAAH-4-B, and BAAH-4-C. Different letters (a–c) between the groups marked a statistically significant difference (*p* < 0.05).

Gel chromatography is an effective technique for purification and screening active peptides from parent protein hydrolysates [26,27]. Seven main peaks, separately named as BAAH-1, BAAH-2, BAAH-3, BAAH-4, BAAH-5, BAAH-6, and BAAH-7, were present in the gel chromatographic spectra of BAAHs (Figure 2A). Both the ACE-inhibitory activity (70.48 ± 2.08% at 1 mg·mL^−1^) and high zinc-chelating capacity (12.30 ± 0.90 mg·g^−1^, Figure 2B) of BAAH-4 were higher than that of the other peaks (*p* < 0.05). After further purification on the Supersil ODS2 column, three peaks, separately named as BAAH-4-A, BAAH-4-B, and BAAH-4-C, were present on the RP-HPLC spectrum of BAAH-4 (Figure 3A). The zinc-chelating ratio of BAAH-4-C (24.44 ± 1.44 mg·g^−1^) and its inhibition activity against ACE (78.28 ± 2.68%) were both higher than those of BAAH-4-A and BAAH-4-B (*p* < 0.05). These results demonstrated that peptide sequences due to both the zinc-chelating ability and inhibition activity against ACE should be identified from BAAH-4-C.

### 2.2. Identification, Screening, and Inhibitory Effect on ACE of BAAH-4-C Peptides

**Figure 4:**
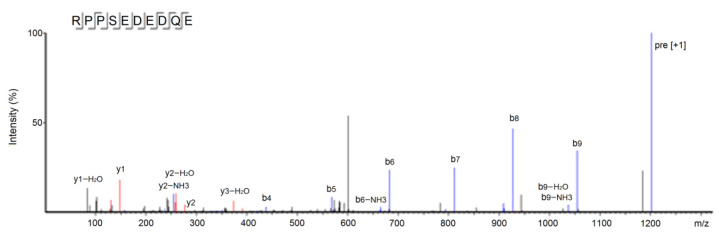
The secondary mass spectrum of peptides RPPSEDEDQE with UPLC-Q-TOF-MS/MS.

**Figure 5:**
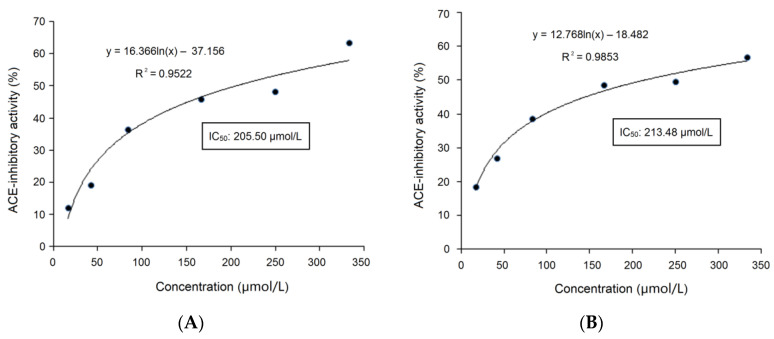
(**A**) The inhibitory effects of RPPSEDEDQE on ACE at different doses, and the fitting equation; and (**B**) the inhibitory effects of RPPSEDEDQE on ACE at different doses, and the fitting equation after the hydrolysis of gastrointestinal enzymes.

The results of ultra-high-performance liquid chromatography and quantum-ultra mass spectrometric determination of BAAH-4-C are shown in Table 1 and Figure 4. Five oligopeptides, Gln-Pro-Pro-Ala-Ala-Ala-Ala-Ala-Ala-Gly-Ala-Gly (QPPAAAAAAGAG), Lys-Thr-Glu-Thr-Gln-Pro (KTETQP), Ser-Pro-Pro-Thr-Ala-Ala-Ala-Ala-Gly-Glu (SPPTAAAAGD), Arg-Pro-Pro-Ser-Glu-Asp-Glu-Asp-Gln-Glu (RPPSEDEDQE), and Thr-Cys-Gly-Ala-Ser (TCGAS) were obtained. However, only RPPSEDEDQE offered potential antihypertension based on the vector machine software score (1.35) calculated by the database AHTpin [28]. Thus, the sequence RPPSEDEDQE was chemically synthesized. The produced RPPSEDEDQE (with purity higher than 99.95%) showed an excellent inhibition activity against ACE (IC_50_: 205.50 μmol·L^−1^, Figure 5A). Additionally, the curve shown in Figure 5A shows that the inhibition activity of RPPSEDEDQE against ACE (*y*) increases with the increase in the dose (*x*), and their relationship conformed to the exponential equation: *y* = 16.366ln(*x*) − 37.156. RPPSEDEDQE showed a higher inhibitory capacity on ACE compared to peptides EKTFLLYSCPHR identified from camel milk (IC_50_: 312 μmol·L^−1^) and FMRWRDRFL isolated from tombul hazelnut (IC_50_: 259.7 μmol·L^−1^) [17,29], but Captopril offered a much higher inhibition capacity on ACE compared with RPPSEDEDQE (*p* < 0.05) [5]. These results suggested that RPPSEDEDQE was a good inhibitor of ACE.

### 2.3. Chelating Capacity toward Zinc Ions and Physicochemical Characteristics

As shown in Table 2, since RPPSEDEDQE contained a high content of Glu and Asp (50%), which have been shown to be ideal binding residues for metal ions [30], it offered an excellent chelating ability toward zinc ions (20.67 ± 3.58 mg·g^−1^). The binding capacity of RPPSEDEDQE toward zinc ions was higher than the other four peptide sequences identified in BAAHs and was equal to that of ethylenediamine tetraacetic acid (EDTA, an excellent metal ions chelator) [31]. Additionally, zinc ions can also be bound to the guanidine of Arg at *N*-terminal, carboxyl group of Glu at the *C*-terminal, and the amide bond near the *N*-terminal of RPPSEDEDQE [2].

Physicochemical characteristics such as hydrophilicity, hydrophobicity, and isoelectric points are important for the antihypertensive effect and application of peptides. However, there are a few studies referring to the physicochemical characteristics of peptides [10,32]. As shown in Table 2, RPPSEDEDQE possessed the highest hydrophilicity (1.85) among the five BAAH-4-C peptides. The hydrophilicity of polypeptide was positively correlated with its polarity which was the main factor affecting the chelation between peptides and zinc ions [32]. Therefore, the high hydrophilicity also reflected the high chelation ability of RPPSEDEDQE toward zinc ions. Since proteins or peptides have a net surface charge of zero and lowest polarity at the isoelectric point (pI), the chelating power of peptides toward metal ions was very low at the pI. Moreover, the polar microenvironment of the peptide may change dramatically at the pI, which affects the binding of the peptide to ACE [33]. Therefore, RPPSEDEDQE and RPPSEDEDQE–zinc chelate should be used away from the pI (3.84, Table 2).

### 2.4. Inhibitory Mechanisms of BAAH Peptides against ACE

#### 2.4.1. Docking Modes of BAAH Peptides toward ACE Molecule

**Figure 6:**
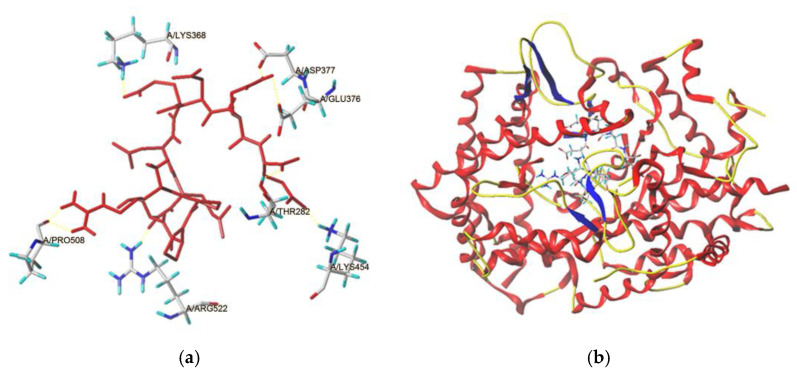
The local diagram (**a**) and global diagram (**b**) of the best docking patterns between RPPSEDEDQE and ACE. The structure of ACE was used PDB: 1O8A model.

In this study, the docking modes of BAAH peptides toward ACE were simulated and virtually screened at the molecular level using a molecular modeling tool. The local diagram and global diagram of the best mode (with the highest T-score) are separately shown in Figure 6a,b. Hydrogen bonds were observed between seven active sites of ACE (Lys368, Asp377, Glu376, Pro508, Thr282, Arg522, and Lys454) and RPPSEDEDQE (Figure 6a and Table 3). Hydrogen bonding is a common but very important force between peptides and ACE. Generally, the length of the hydrogen bond was inversely proportional to the force between the peptides and ACE [5,7]. Thus, the short length of the hydrogen bonds between ACE and RPPSEDEDQE (1.94–2.91 Å) suggested that the binding power of RPPSEDEDQE toward ACE was strong. More importantly, the T-score for the best docking mode of BAAH peptides toward ACE (8.06) was above the threshold (6.0), reflecting a strong binding power of RPPSEDEDQE toward ACE. Additionally, hydrophobic interactions were observed between RPPSEDEDQE and fifteen residues in ACE (Table 3). These results were consistent with the high inhibitory effect against ACE (IC_50_: 205.50 μmol·L^−1^).

In addition, hydrophobic interactions were also observed between RPPSEDEDQE and His387, a main residue of the zinc tetrahedral coordination in ACE (Table 3). The effects of peptides on the zinc tetrahedral coordination may change ACE’s catalytic function [3]. The results in Figure 6 and Table 3 demonstrated that the inhibitory mechanism of BAAH peptides against ACE was mainly the binding of RPPSEDEDQE with ACE, especially the interactions between RPPSEDEDQE and the zinc tetrahedral coordination.

#### 2.4.2. Inhibition Kinetics on ACE

**Figure 7:**
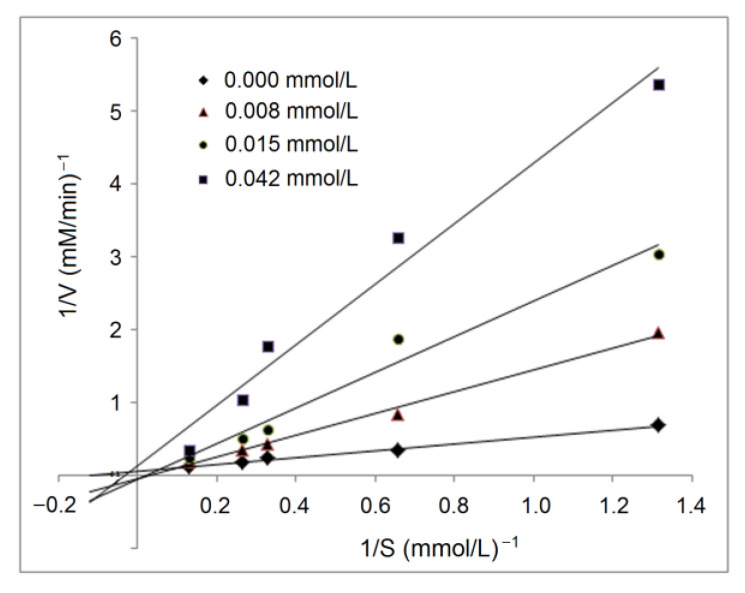
Lineweaver–Burk chart for inhibitory capacity of RPPSEDEDQE toward ACE.

The active residues of ACE that RPPSEDEDQE can dock with via hydrogen bond or hydrophobic interactions did not locate in the binding center of ACE [4], indicating that RPPSEDEDQE offered an uncompetitive inhibitory effect against ACE, which was evidenced by the results of the inhibition kinetics test, as shown in Figure 7. As shown in the Lineweaver–Burk diagram of ACE, the *K_m_* increased with the increasing RPPSEDEDQE dose, but the maximum velocity (*V_max_*) decreased when the RPPSEDEDQE dose increased, which was the typical effect of noncompetitive inhibitors [7]. Peptides such as HLNVVHEN and PGSGCAGTDL identified from walnut protein, and LVRYYLLVR from hazelnut protein all showed noncompetitive ACE inhibitory patterns [14,17].

#### 2.4.3. The Binding Mode of RPPSEDEDQE to Zinc Ion

**Figure 8:**
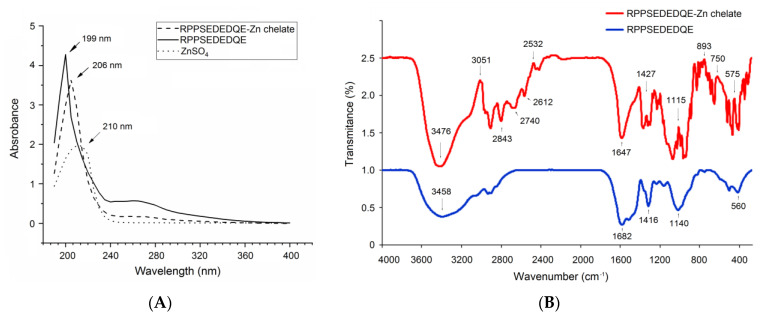
Ultraviolet wavelength scanning profiles (**A**) and Fourier-transformed infrared spectroscopic diagrams (**B**) of RPPSEDEDQE zinc ligands. Pure RPPSEDEDQE was used as comparison.

In the current study, ultraviolet wavelength scanning and Fourier-transformed infrared spectroscopy were both employed to analyze the binding mode of RPPSEDEDQE to zinc ion. The ultraviolet adsorption peak of RPPSEDEDQE moved from 199 nm to 206 nm after the zinc chelation (Figure 8A), demonstrating the combination between zinc ions and RPPSEDEDQE [34]. Chelation with zinc ions can cause electronic transitions of peptides’ chromophoric groups, thereby resulting in a red- or blue shift of the ultraviolet adsorption peak of peptides [35].

As shown in Figure 8B, after chelation with zinc ions, the RPPSEDEDQE–zinc chelate showed a different FT-IR spectrum compared with the pure RPPSEDEDQE, suggesting that zinc ions have been linked with RPPSEDEDQE. After the coordination of RPPSEDEDQE with zinc ions, the absorption peaks presented at 3458 cm^−1^ (corresponding to a stretching of –N–H), 1682 cm^−1^ (indicative of the vibration of –C–N), and 886 cm^−1^ (representing the deformation of the amide IV band) [36,37] have separately moved to 3476 cm^−1^, 1647 cm^−1^, and 893 cm^−1^, evidencing the binding between zinc ions and the peptide linkage or amino group of RPPSEDEDQE [33]. Moreover, the redshift from 1416 to 1427 cm^−1^ reflected that an asymmetric bend had happened on –C–O, showing the binding of zinc ions with the carboxyl group of RPPSEDEDQE [32]. Thus, the results in Figure 8A,B show that RPPSEDEDQE can provide multiple binding sites for zinc ions.

In short, the inhibitory mechanisms of RPPSEDEDQE against ACE may include binding with seven active residues of ACE, and coordinating with His387 or the zinc ion of the zinc tetrahedral coordination.

### 2.5. Stability of RPPSEDEDQE

After gastrointestinal digestion, the inhibitory effect of RPPSEDEDQE on ACE (*y*) at different doses (*x*) is shown in Figure 5B. The equation fitting to this curve was *y* = 12.768ln(*x*) − 18.482, with an IC_50_ value of 213.48 μmol·L^−1^, which was equal to that of untreated RPPSEDEDQE (205.50 μmol·L^−1^, Figure 5A). Moreover, as shown in Table 2, after gastrointestinal digestion, the chelating activity of RPPSEDEDQE toward zinc ions was 18.55 ± 0.95 mg·g^−1^, which was not different from that of untreated RPPSEDEDQE (*p* > 0.5). It can be obtained that the zinc-chelating ability and ACE-inhibitory capacity of RPPSEDEDQE were both not significantly reduced by the hydrolysis of gastrointestinal proteases (*p* > 0.5).

### 2.6. Antihypertension In Vivo of RPPSEDEDQE and RPPSEDEDQE–Zn Complexes

**Figure 9:**
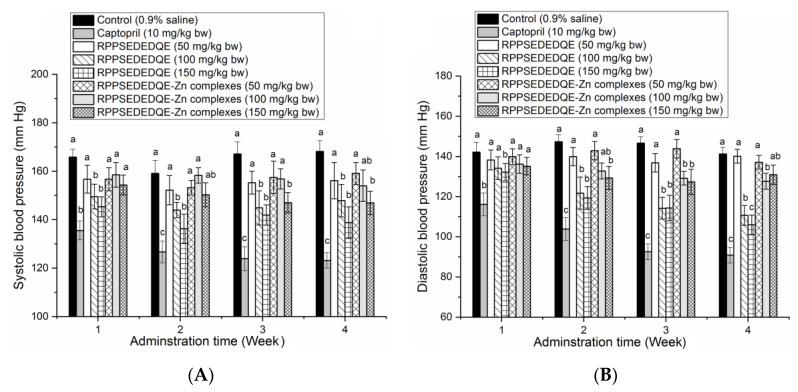
Efficiency of RPPSEDEDQE and RPPSEDEDQE–Zn complexes on the systolic blood pressure (**A**) and diastolic blood pressure (**B**) of spontaneously hypertensive rats. Different letters (a–c) between the groups marked a statistically significant difference (*p* < 0.05).

As shown in Figure 9A, a moderate to high dose of RPPSEDEDQE (100–150 mg·kg bw^−1^) significantly decreased the systolic blood pressure of spontaneous hypertensive rats (SHR) after 1 week of oral administration (*p* < 0.05); in comparison, a high dose of RPPSEDEDQE–Zn complexes obviously reduced the systolic blood pressure of SHR after 3 weeks’ administration (*p* < 0.05). A low dose of RPPSEDEDQE (50 mg·kg bw^−1^) and low to middle dose of RPPSEDEDQE–Zn complexes (50–100 mg·kg bw^−1^) did not show any lowering effect on the systolic blood pressure of SHR. A similar trend was observed in their effects on the diastolic blood pressure of SHR (Figure 9B).

RPPSEDEDQE remarkably lowered the diastolic blood pressure of SHR at 150 mg/kg body weight after one week of oral administration (*p* < 0.05). RPPSEDEDQE–Zn complexes with a high dose (150 mg·kg bw^−1^) decreased the diastolic blood pressure after two weeks of administration. However, a low dose of RPPSEDEDQE and RPPSEDEDQE–Zn complexes did not offer any reducing effect on the diastolic blood pressure of SHR. At the same dose, RPPSEDEDQE’s reducing effects on the systolic blood pressure and the diastolic blood pressure were both better than that of RPPSEDEDQE–Zn complexes. Moreover, the reducing efficiency of RPPSEDEDQE and RPPSEDEDQE–Zn complexes on the diastolic and systolic blood pressure were both much lower than that of the positive control (Captopril, a famous antihypertensive drug) (*p* < 0.05) [7].

In short, RPPSEDEDQE was a dose-dependent blood-pressure lowerer, and chelation with zinc ions may hinder its antihypertensive efficiency. The results in Figure 7 showed that the amino group, carboxyl group, and amide bond of RPPSEDEDQE could all chelate with zinc ions. The coordination with zinc ions may affect the interactions between RPPSEDEDQE and ACE, especially the zinc tetrahedral coordination, consequently lowering the antihypertensive effect of RPPSEDEDQE [35]. Therefore, the results of this study suggested that RPPSEDEDQE was not suitable to use as a zinc supplement and blood-pressure-lowering agent at the same time.

## 3. Discussion

To the best of our knowledge, this is the first study to simultaneously investigate the capacities of bitter almond albumin peptides to inhibit ACE and bind with zinc ions. Previous studies evidenced that thermolysin could quickly cleave the amide bonds linked with hydrophobic amino acid residues, with the released hydrophobic amino acid residues playing an important role in ACE inhibition; whereas papain cleaves multiple sites of polypeptide chains, especially peptide bonds linking with acidic amino acid residues (Glu and Asp), Arg, and aromatic amino acids which was helpful for the release of peptide sequences due to the metal ion-chelating capacity and/or inhibiting ability on ACE [23,38]. Thus, to release the capacities of peptides to inhibit ACE and chelate with zinc ions, bitter almond albumin was hydrolyzed by papain and thermolysin in the current study. Zhang et al. [25] used dual enzymes (flavorzyme and alcalase with a mass ratio of 1:2) to hydrolyze almond amandin, and the hydrolysis degree and zinc-chelating capacity of the hydrolysates were 32.67% ± 4.56% and 14.32 ± 1.32 mg·g^−1^, respectively. These results indicated that papain and thermolysin rather than flavorzyme combined with alcalase could isolate the peptide sequences of the zinc-chelating ability and ACE-inhibitory ability from bitter almond albumin polypeptide chains. A previous study found that papain probably prefers to hydrolyze peptide bonds linking with acidic amino acid residues (Glu and Asp), which was helpful for the release of peptide sequences owing to the metal ion-chelating capacity [17]. Moreover, BAAHs offered a higher inhibiting ability against ACE (53.99 ± 4.62%) than that of wild almond protein hydrolysates (18.26%) at 1 mg·mL^−1^ [16].

A previous study showed that *C*-terminal tripeptides are crucial for the inhibition ability of peptides against ACE. *C*-terminal hydrophobic tripeptides are accepted as the main reason for the excellent inhibiting ability of peptides on ACE [29]. In contrast, the *C*-terminal tripeptides of RPPSEDEDQE (Asp, Gln, and Glu) were all hydrophilic, but it offered a relatively high ACE-inhibitory activity (205.50 μmol·L^−1^). The main reason was perhaps that the Gln and Glu residues both had a relatively high binding affinity with the active sites of ACE. Peptides with Glu and Gln in the *C*-terminal tripeptide such as ADMIET identified in *Lepidium meyenii*, QTPHQ, and ELHPQ derived from canary seeds also showed high ACE-inhibitory activities [29,30]. Moreover, the *N*-terminal tripeptides of RPPSEDEDQE were Arg, Pro, and Pro which have been shown to have a strong affinity to ACE [7,31]. The peptides RTVFDGEL and RADVFNPR derived from palm kernel glutelin-1 with Arg at the *N*-terminal exhibited an excellent ability to restrain ACE [27].

Recently, most studies have been focusing on the interaction between the peptides and binding sites of ACE, but few data referred to the effects of peptides on the zinc tetrahedral coordination. The results in Figure 6 and Figure 7 evidenced that RPPSEDEDQE can interact with the residue His387 of the zinc tetrahedral coordination and provide multiple chelating sites for zinc ions. Li et al., and Zarei et al., found that symbiotic peptides and palm oil kernel peptides could affect the zinc tetrahedral coordination and offered high ACE inhibition abilities [5,26]. However, the effect of RPPSEDEDQE on the zinc tetrahedral coordination in ACE needs further work.

Although previous studies showed that oligopeptides were sensitive to the protease existing in the gastric or intestinal regions [10,12], RPPSEDEDQE exhibited a relative stability against the hydrolysis of gastrointestinal proteases. One of the reasons may be its high content of Pro residue. It was shown that the rigid ring structure of Pro was helpful for the resistance of peptides to enzymatic digestion [13]. The peptides PPSEPTKL and VEPFPLF that were rich in Pro residue and isolated from bighead carp were found to be stable under the hydrolysis of gastrointestinal enzymes [36]. However, to investigate the changes in the RPPSEDEDQE structure under the hydrolysis of gastrointestinal enzymes, more work is needed.

The results of the current study showed that zinc chelation reduced the in vivo antihypertensive effect of RPPSEDEDQE. It was shown that Mung bean-derived peptides PGSGCAGTDL, camellia peptide GYGYNY, and LYSPLLKYFD from rice bran had dose-dependent antihypertensive effects on SHR, but the addition of zinc ions can reduce their antihypertension [35,39,40]. Zinc chelation may change the coordination of peptides toward ACE, resulting in lower ACE-inhibitory activity and antihypertension [4]. However, Li et al. [5] found that the ACE inhibition ability of symbiotic peptide KYPHVF was not significantly reduced after zinc chelation. Therefore, the specific effect of zinc chelation on the antihypertensive activity of peptides needs further work.

## 4. Materials and Methods

### 4.1. Materials and Reagents

Bitter almonds were purchased in Xinglin Garden, Taigu, China. Thermolysin (USP, 1:100,000 U·g^−1^, where one unit (U) will hydrolyze casein to produce color equivalent to 1.0 μmol of tyrosine per min at pH 7.5 and 37 °C), papain (USP, 1:10,000 U·g^−1^), pancreatin (USP, 1:3000 U·g^−1^), and pepsin (USP, 1:100,000 U·g^−1^) were provided by Yunheng Biotechnology Company, Suzhou, China. ACE and *N*-hippuryl-L-histidyl-L-leucine were purchased from Sigma (St. Louis, MO, USA). Sodium hydroxide, ZnSO_4_·7H_2_O, and other analytical grade chemicals were purchased from Suma Chem. Co. (Shijiazhuang, China).

### 4.2. Extraction and Proteolysis of Bitter Almond Albumin

Bitter almonds were washed and dried at 50 ± 1 °C for 17 h. The shells of the bitter almonds were removed, and the kernel was ground, and then sieved using a JL-003 sieve (60–mesh, Jujin Screen Factory, Xinxiang, China) [20]. Next, 50 g of bitter almond was mixed with 450 mL of petroleum ether (60–90 °C), and shaken in a THZ-82AB thermostatic oscillator (Nabeichao Shaker Co., Foshan, China) at 40 r/min and 25 °C for 4 h. After filtration, the mud left on the filter paper was dried at 45 ± 1 °C for 3 h to obtain defatted bitter almond. Afterward, the defatted bitter almond (10 g) was thoroughly mixed with 400 mL of distilled water (dH_2_O), and incubated in the THZ-82AB thermostatic oscillator (with a rotation speed of 210 r/min) at 45 ± 1 °C for 200 min. Then, the mixture was filtered, and the percolate was centrifuged at 13,000× *g* and 5 °C for 25 min. The supernatant was placed in membranes with a cut-off mass of 7.0 kDa and dialyzed against dH_2_O at 4 °C for 36 h. A freeze-dryer (Tingya Centrifuge Factory, Wuhan, China) was employed to dry the dialysis solutions in the membranes, and bitter almond albumin (SAA) was obtained.

As per the method of Zhang et al. [25] with some modifications, in a THZ-82AB thermostatic oscillator with a rotation speed of 170 r/min, SAA (4 g·200 mL^−1^ dH_2_O) was hydrolyzed by papain (0.1 g) at 50 °C and pH 7.5 for 120 min. Next, the temperature was decreased to 37 °C, and thermolysin (0.1 g) was added, the proteolysis reaction was continued for 120 min. To maintain the pH value of the reaction solution at 7.5 ± 0.1, the reaction solution was adjusted with dilute 1 mol·L^−1^ of HCl or 1 mol·L^−1^ of NaOH every five minutes. Afterward, the hydrolysis solution was heated in boiling water for 5 min. After centrifugation at 10,480× *g* for 22 min, the supernatant was freeze-dried with a lyophilizer (Tingya Centrifuge Factory, Wuhan, China) to obtain bitter almond albumin hydrolysates (BAAHs). Additionally, the hydrolysis degree of bitter almond albumin was determined using the method of Nielsen et al. [41].

### 4.3. Chromatographic Separation of BAAHs

The BAAH (0.2 g/100 mL Milli-Q water) was first separated on a gel column (size: 8 × 100 cm, Xihu, Shanghai, China) chromatography filled with Sephadex G-25. The mobile phase was Milli-Q water with an elution speed of 3.2 mL every minute, and the monitored wavelength was 220 nm [28]. The corresponding peak fractions were collected, pooled, freeze-dried, and their inhibition ability against ACE was measured as per the *N*-hippuryl-L-histidyl-L-leucine method [30]. The further separation of subfractions was conducted using an LC-20AT reverse-phase high-performance liquid chromatography instrument (RP-HPLC, Shimadzu, Tokyo, Japan). The subfraction that offered the highest inhibiting ability against ACE was loaded on a Supersil ODS2 chromatographic column (Size: 2.1 × 30 mm, 5 μm; Eilt, Suzhou, China) and eluted by acetonitrile (elution A) and trifluoroacetate solution (1 mL·1000 mL^−1^ ultrapure water, elution B). The RP-HPLC separation was conducted with binary gradient elution at 35 °C for 24 min [7]. The gradient concentration of acetonitrile against elution time was 0–3 min, 5% A; 4–8 min, 5–10% A; 9–15 min, 10–35% A; 13–24 min, 35% A. The corresponding peak fractions were collected and separately freeze-dried, and the fraction offering the highest inhibition ability against ACE was subjected to peptide sequence analysis.

### 4.4. Inhibition Activity and Kinetics against ACE

The *N*-hippuryl-L-histidyl-L-leucine method was utilized for the determination of the inhibiting capacity of BAAH peptides toward ACE [30]. In a glass test tube, 0.2 mL of ACE solution (dissolved in boric acid buffer, pH 8.3) and 0.2 mL of BAAH peptide aqueous solution (1 mg·mL^−1^) were added. The mixture in the glass tube was shaken in a water bath at 143 r·min^−1^ and 37 °C. Ten minutes later, 0.65 mL of *N*-hippuryl-L-histidyl-L-leucine (5 mmol·L^−1^, dissolved in boric acid buffer, pH 8.3) was added. After continuously shaking for 1 h, 0.75 mL of dilute hydrochloric acid was added into the tube. Next, hippuric acid produced in the reaction solution was extracted by ethyl acetate (1.25 mL). The hippuric acid solution (the supernatant) was sucked out with a pipette (DG-1, Dragon TopPette, Beijing, China) and heated at 70 °C to evaporate the ethyl acetate. The residual hippuric acid was resolved in distilled water, and its absorbance at 228 nm was read. The inhibition capacity of BAAH peptides against ACE was the percentage of the absorbance reduction value at 228 nm to the absorbance of the control group (without BAAH peptide). The concentration of BAAH peptides was defined as IC_50_ when the activity of ACE was inhibited by half. Captopril was used as the positive control.

Furthermore, the Lineweaver–Burk plot of ACE against different concentrations of *N*-hippuryl-L-histidyl-L-leucine (0.13, 0.26, 0.33, 0.66, and 1.32 mmol·L^−1^) was drawn [30]. Both *K_m_* (Michaelis–Menten constant) and the max reaction velocity (*V_m_*) were calculated to analyze the inhibition kinetic of BAAH peptides (0.008, 0.015, and 0.042 mmol·L^−1^) against ACE.

### 4.5. Chelating Ability toward Zinc Ions

The chelating ability of BAAH peptides toward zinc ions was measured using the 4-(β-Pyridinazo)-resorcinol method following the same procedures proved by Ke et al. [36]. In short, 0.75 mmol·L^−1^ of ZnSO_4_ (dissolved in 0.2 mmol·L^−1^ of HEPES-KOH buffer) and 10 mmol·L^−1^ of 1,4-Dithiothreitol were added into a glass tube. The tube was incubated in the THZ-82AB thermostatic oscillator (195 r·min^−1^ and 25 °C). Five minutes later, the zinc concentration in the glass tube was measured and read as *C*_0_. Next, the BAAH peptides aqueous solution (0.8 mg·mL^−1^) was added and the mixture was continuously shaken for another 15 min. Then, the zinc concentration in the glass tube was measured and read as *C_t_*. The chelating ability of BAAH peptides toward zinc ions was calculated as follows, and ethylenediamine tetraacetic acid (EDTA) was used as the positive control.

(1) $$Chelating\;ability\;towards\;zinc\;ions\;(mg\cdot g^{-1})\;=\;{(C}_{0}-C_{t})\;\times \;V/m$$

where *V* and *m* represent the volume of the reaction solution (mL) and the mass weight of BAAH peptides (g), respectively.

### 4.6. Amino Acid Sequence Analysis, Validation, and Screening

First, the lyophilized BAAH subfractions isolated by RP-HPLC were further analyzed on a LaChrom II C_18_ column (3 × 250 mm, 1.9 μm) coupled to an ultra-high-performance liquid chromatography system (ChromasterUltra Rs, HITACHI, Tokyo, Japan) using the same procedures of Ruan et al. [7]. Next, the quadrupole Orbitrap tandem spectrometric determination was conducted on an ACQUITY TQD quantum-ultra mass spectrometer (Waters, Milford, MA, USA) coupled with Peak de novo 8.5 software (BSI, Waterloo, ON, Canada). The specific parameters for the electrospray ionization operation were ion source gas of 60 psi; spray negative voltage of 4300 and positive voltage of 5300 V; mass range from 120 to 1600 *m*/*z*; and AGC target value of 1 × 10^5^ [33]. Validation of the BAAH amino acid sequences identified was carried out using the database from the National Biotechnology Information Center (Bethesda, MD, USA). Moreover, the BAAH amino acid sequences identified were in silico screened on the basis of the vector machine software score (SVMS) using the database AHTpin [27]. BAAH sequences that had an SVMS higher than zero were accepted as potential antihypertensive peptides [42].

### 4.7. Chemical Synthesis, Chelating Capacity toward Zinc Ions, and Physicochemical Characteristics of Potential Antihypertensive Sequences

BAAH amino acid sequences with potential antihypertension were synthesized following the chemical solid procedures in Gangyan Biotech. Co. (Dalian, China). The chemical-synthesized peptides were subjected to inhibition ability against ACE and chelating capacity toward zinc ions determinations. Moreover, the physicochemical characteristics of BAAH amino acid sequences were calculated using in silico tools with database AHTpin [27].

### 4.8. Docking Modes of BAAH Peptides toward ACE Molecule

Interactions of BAAH peptides toward ACE were determined using a molecular modeling tool (SERFLUX-DOCK SCREEN, SYLYB 2.2.2, Treops Company, Jefferson City, MO, USA) with a three-dimensional structure of ACE (PDB-108A, from Protein Bank) as the docking model. Ligands of ACE and BAAH peptides were virtually screened using multiple scoring functions including Kuntz-D Score, FleX-Chem Score, and Martin-PMF Score, and total score of virtual evaluation (T-score), and consistency score (C-score) were used as the main evaluation index. The T-score of acceptable docking modes should be higher than 6.0. The interaction forces between the interfacing sites of ACE and BAAH peptides including hydrogen bonds and hydrophobic interactions were investigated [7,17].

### 4.9. Ligand Patterns of Zinc Ions with BAAH Peptides

First, BAAH peptides and zinc ion ligands were prepared with ZnSO_4_·7H_2_O as the zinc ion donor, using the same procedures of Zhang et al. [37]. BAAH peptides were chelated with ZnSO_4_·7H_2_O at a mass ratio of 1:7. The reaction temperature and pH were 52 °C and pH 5.8, respectively [33]. The reaction solution was centrifuged and the supernatant was further precipitated by anhydrous ethanol. Then, the precipitate was freeze-dried and the BAAH peptides’ zinc chelation was obtained.

Second, the obtained BAAH peptides’ zinc chelation was subjected to ultraviolet wavelength scanning analysis and Fourier-transformed infrared spectroscopy (FT-IR) determination, respectively. In short, ultraviolet wavelength scanning was conducted using an UV759CRT ultraviolet wavelength scanner (Jingke Instrument Lit. Co., Shanghai, China). The range of the scanned wavelength was from 190 to 400 nm [33]. Simultaneously, 3 mg of the BAAH peptides’ zinc chelation and dry KBr (0.01 g) were pressed into a translucent sheet. The sheet was used for FT-IR analysis on an FT-730 spectrometer (Jinke Instrument Factory, Shanghai, China) with wavenumbers from 4000 to 400 cm^–1^ [32].

### 4.10. Stabilities of BAAH Peptides

In a stirring (115 r·min^−1^) water bath at 37 °C, the BAAH peptides’ aqueous solution (5 mg/mL, 10 mL) was added into a glass test tube, and the pH value was adjusted to 2.00 ± 0.05 with dilute hydrochloric acid solution (0.5 mol·L^−1^) [13]. Three minutes later, 1.15 mg pepsin (USP, 1:10,000 U/g) was added into the glass tube and mixed thoroughly, and then stirred in the water bath for ninety minutes. Next, the pH value was adjusted to pH 7.00 ± 0.05 with dilute sodium hydroxide solution (0.1 mol·L^−1^), and then 2.25 mg pancreatin (USP, 1:3000 U·g^−1^) was added. The hydrolysis reaction was continued for another 150 min. Then, the glass tube was heated in boiling water to stop the digestion reaction. Afterward, both the chelating ability toward zinc ion, and the inhibition activity of BAAH peptides against ACE were determined using the 4-(β-Pyridinazo)-resorcinol method and *N*-hippuryl-L-histidyl-L-leucine method, respectively [30,36]. Untreated BAAH peptides were used as the blank control.

### 4.11. Antihypertension In Vivo

The antihypertensive effect was measured as per the modified method of Dong et al. [40]. Male spontaneously hypertensive rats (SHR, 10 weeks, 220 ± 15 g body weight, from Beijing Medicine Yaoli Biotechnology Co., Ltd., Beijing, China) were housed in colony cages maintained at 25 ± 1 °C and 45–55% relative humidity with a 12 h light/dark cycle. Tap water was freely available. The rats were randomly divided into eight groups: negative group, positive group, BAAH peptide low-, middle-, and high-groups, and BAAH–Zn complexes low-, middle-, and high-groups. Each group had four rats. The rats in the negative control group and positive group were gastric intubated with NaCl (0.9%) and Captopril (15 mg/kg/bodyweight once daily), respectively; while the rats in the high-, middle-, and low-dosage groups were orally administrated with 200, 100, and 50 mg of MBAH peptides (or peptide–Zn complexes)/kg/bodyweight once daily, respectively. The oral administration was continued for 28 days. The diastolic and systolic blood pressures of the rats were measured every 7 days using the tail-cuff method with a ZL-620-F noninvasive blood-pressure apparatus (Anhui Yaokun Biotechnology Co., Ltd., Hefei, China). Each rat was measured at least three times. The weight and heart rate of the rats were determined at the same time. This experimental work was approved by the Institutional Animal Care and Use Committee, Shanxi University of Chinese Medicine (No. SNUA230311002). All animals received human care according to the Guidelines Manual for the Care and Use of Laboratory Animals.

### 4.12. Statistical Analysis

The entire test was repeated three or more times and one-way variance analysis and Duncan’s multiple comparison were employed to analyze the difference among results. The difference was considered as significant when the *p* value was less than 0.05.

## 5. Conclusions

A zinc ion ligand RPPSEDEDQE offering an inhibitory effect on ACE (IC_50_: 205.50 μmol·L^−1^) was isolated from BAAHs using an in vitro test combined with in silico methods. The binding modes of RPPSEDEDQE to the ACE molecule perhaps include three ways: (i) the formation of hydrogen bonds between RPPSEDEDQE and seven active residues of ACE; (ii) RPPSEDEDQE bound to fifteen active residues of ACE via hydrophobic interactions; and (iii) RPPSEDEDQE link with the residue His387 or zinc ion of the zinc tetrahedral coordination in ACE. Inhibitory kinetics analysis evidenced that RPPSEDEDQE was a noncompetitive inhibitor of ACE, because it cannot bind with any central pocket of ACE. RPPSEDEDQE possessed a high hydrophilicity (1.85), but RPPSEDEDQE and RPPSEDEDQE–zinc chelate should be used away from the pI (3.84). Moreover, the zinc-chelating ability and ACE-inhibitory capacity of RPPSEDEDQE were both resistant to the hydrolysis of gastrointestinal enzymes. RPPSEDEDQE (100–150 mg·kg bw^−1^) significantly reduced the systolic and diastolic blood pressure of SHRs, but chelation with zinc ions decreased its antihypertensive efficiency. These results indicate that bitter almond albumin peptides may be used for antihypertension. The results of the current study, especially the studies on physicochemical characterization, safety, and effect on the zinc tetrahedral coordination in ACE, can provide a new perspective for studying the action mechanism and application of antihypertensive peptides. 

## Figures and Tables

**Table 1:** Identification, in silico screening, chelating ability toward zinc ions, inhibitory effect against ACE, and stability of sequences from bitter almond albumin hydrolysates.

Sequences	RPPSEDEDQE	KTETQP	TCGAS	SPPTAAAAGD	QPPAAAAAAGAG
Molecular mass (Da)	1201.29	535.61	437.52	857.00	766.96
Matched sequence in *Semen Armeniacae Amarum* ^a^	P.RPPSEDEDQE.Y	R.KTETQP.-	S.TCGAS.S	F.SPPTAAAAGD.M	G.QPPAAAAAAGAG.R
Vector machine software score ^b^	1.35	0.90	−0.58	−0.96	−0.52
Antihypertension predict ^c^	AHP	Non-AHP	Non-AHP	Non-AHP	Non-AHP
Inhibitory effect against ACE (IC_50_: μmol·L^−1^)	205.50	ND	ND	ND	ND
Inhibitory effect against ACE after gastrointestinal digestion (IC_50_: μmol·L^−1^)	213.48	ND	ND	ND	ND
Chelating ability toward zinc ions (mg·g^−1^)	20.67 ± 3.58 d	19.30 ± 1.46 e	6.44 ± 0.64 f	2.16 ± 0.45 g	1.14 ± 0.01 g
Chelating ability toward zinc ions after gastrointestinal digestion (mg·g^−1^)	18.55 ± 0.95	ND	ND	ND	ND

^a^ From National Center for Biotechnology Information (NCBI); ^b^ Vector machine software score and potential antihypertension were in silico screened with database AHTPIN; ^c^ AHP: antihypertensive peptide. ND: not measured. Different letters (d–g) between the groups marked a statistically significant difference (*p* < 0.05).

**Table 2:** In silico analysis of physicochemical characteristics of bitter almond albumin peptides using AHTPIN database.

Peptides	RPPSEDEDQE	KTETQP	TCGAS	SPPTAAAAGD	QPPAAAAAAGAG
Acidic amino acid content (%)	50.00%	16.67%	0.00%	10.00%	0.00%
Hydrophobic amino acid content (%)	20.00%	16.67%	20.00%	60.00%	75.00%
Hydrophobicity	−0.61	−0.09	0.00	−0.01	0.18
Amphiphilicity	0.75	1.59	0.00	0.00	0.00
Hydrophilicity	1.85	−0.66	−0.32	0.09	−0.35
Isoelectric point	3.84	7.25	5.85	3.80	5.88

**Table 3:** Virtual evaluation on the docking modes of RPPSEDEDQE with ACE, and interaction forces in the best mode.

Ligand	T-Score	C-Score	Interaction Force	Active Sites of ACE and Hydrogen Bond Length
RPPSEDEDQE	8.06	5	Hydrogen bond	Lys368: 1.94 Å; Asp377: 2.91 Å; Glu376: 2.47 Å; Pro508: 2.03 Å; Thr282: 1.97 Å; Arg522: 2.12 Å; Lys454: 2.81 Å
			Hydrophobic interactions	His353, Cys352, Tyr523, Ala354, Ser355, Phe457, Phe527, Asp415, Pro163, Gln369, Cys370, Glu376, Asn374, Ala170, Asn167

T-score is the total score of virtual evaluation, and C-score means the consistency score.

## Data Availability

The data that support the findings of this study are available from the corresponding author upon reasonable request.

## Sample Availability

Samples of the bitter almond albumin are available from the authors.
